# Malignant Hidradenocarcinoma of the Axilla

**DOI:** 10.7759/cureus.7091

**Published:** 2020-02-24

**Authors:** Evan P Johnson, Jonathan Keyes, Vania Zayat, Jeremy Caudill

**Affiliations:** 1 Orthopaedics, University of Central Florida College of Medicine, Orlando, USA; 2 Internal Medicine, University of Central Florida College of Medicine, Orlando, USA; 3 Pathology, University of Central Florida College of Medicine, Orlando, USA; 4 Orlando Veterans Affairs Medical Center, Orlando, USA; 5 Surgery, Flagler Hospital, St. Augustine, USA

**Keywords:** hidradenocarcinoma, general surgery, rare cancers, dermatology, pathology, oncology, skin cancers

## Abstract

Malignant hidradenocarcinoma is a very rare and highly aggressive primary skin neoplasm that arises in the eccrine sweat glands. Diagnosis is typically made with histopathological evaluation after excisional biopsy. Reports of this tumor are scarce in the literature, thus making its characterization and management particularly challenging.

A 71-year-old male presented in the clinic with swelling of the left lateral axilla on routine dermatological examination. Clinically, the lesion was suspected to be a capillary hemangioma. Upon surgical excision, the specimen was diagnosed as malignant hidradenocarcinoma based on histological characterization with immunohistochemical staining. Subsequent wide excision with sentinel lymph node biopsy was performed, which came back negative for residual tumor and metastasis.

Due to the low incidence of this cancer and the markedly poor prognosis, accurate diagnosis of these tumors is highly important. Wide excisional biopsy and sentinel lymph node biopsy appear to be the most common initial treatment plans based on the available literature. With high rates of recurrence and metastasis, there remains the need to characterize effective adjuvant therapy for the post-operative management of hidradenocarcinoma.

## Introduction

Hidradenocarcinoma is an exceedingly rare and particularly aggressive malignancy of the eccrine sweat glands. The prognosis is poor with a five-year survival rate of 30% and high rates of metastatic disease and recurrence [1,2]. Due to its low incidence, the management of hidradenocarcinoma has not been well described in the current literature. Adjuvant chemotherapy and radiation are controversial, and the need remains to elucidate a clear and effective treatment regimen beyond wide excision [3]. Here, we present a case of hidradenocarcinoma of the axilla that was managed with wide surgical excision and sentinel lymph node biopsy. Currently, the patient is undergoing radiation therapy, as well as anti-androgen hormonal treatment. Written consent was obtained from the patient.

## Case presentation

A 71-year-old Caucasian male with no significant past medical history presented with a firm, non-tender, well-circumscribed, subcutaneous, non-ulcerated, slightly erythematous mass of the left lower axilla on routine dermatological examination. The mass had been slowly increasing in size for approximately six months, with a gross measurement of 1.5 x 1.0 cm at the initial visit. The patient was asymptomatic with no regional lymphadenopathy noted on physical examination. The remainder of the physical examination was within normal limits. The patient is a lifetime non-smoker and does not drink alcohol.

**Figure 1:**
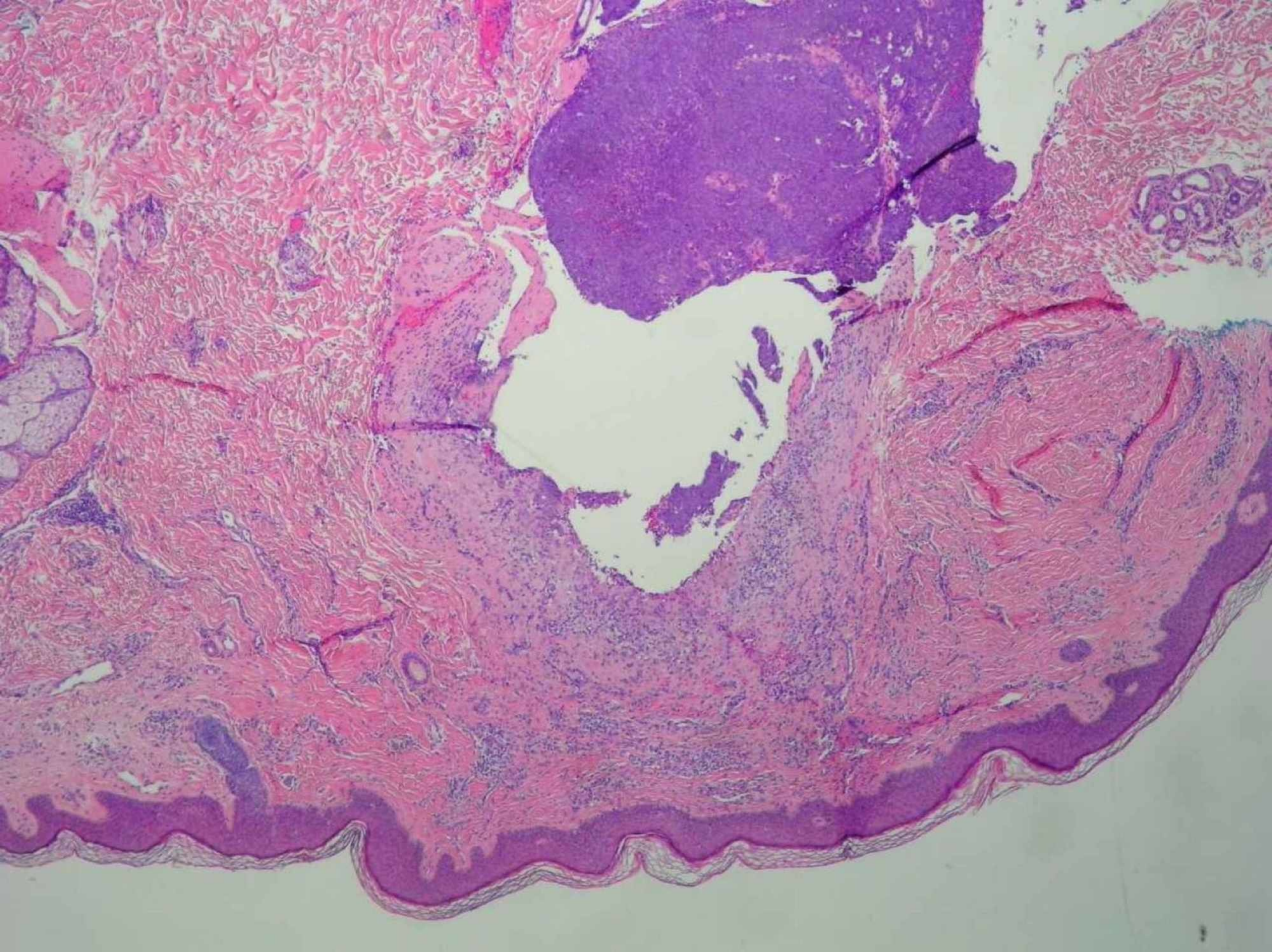
Histopathology of Tumor Specimen Mag. 4x A photomicrograph of hidradenocarcinoma showing skin with an underlying ill-circumscribed lobulated infiltrating mass extending to the dermis (hematoxylin and eosin [H&E] stain, mag. 4x).

**Figure 2:**
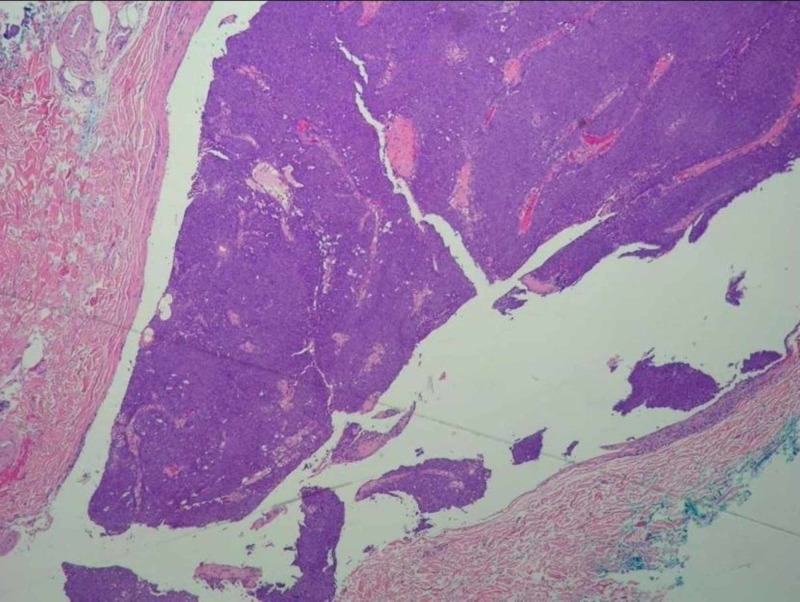
Histopathology of Tumor Specimen Mag. 20x A photomicrograph of hidradenocarcinoma showing sheets of basophilic cells deep in the dermis approaching the inked surgical margin (hematoxylin and eosin [H&E] stain, mag. 20x).

**Figure 3:**
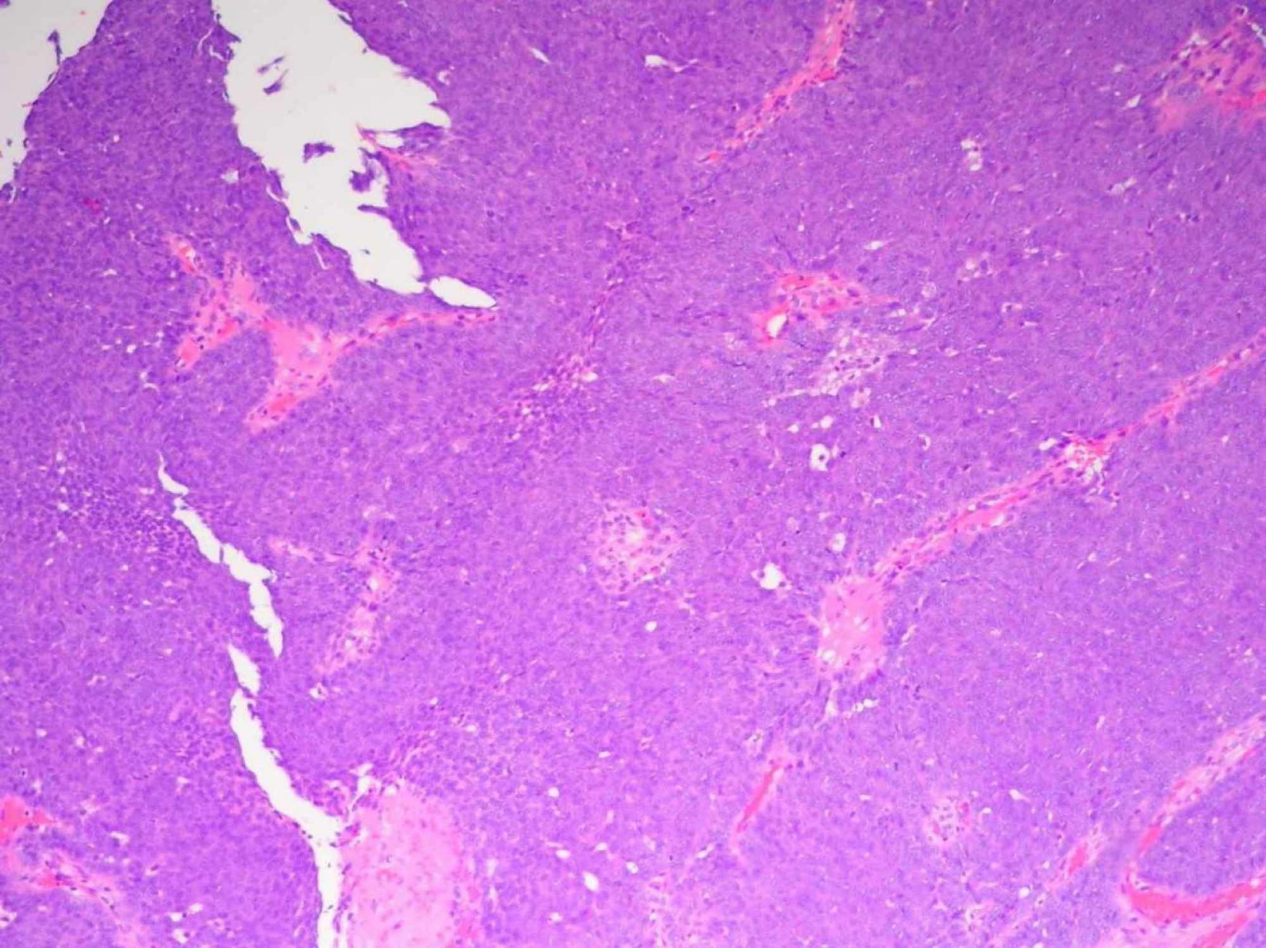
Histopathology of Tumor Specimen Mag. 40x A photomicrograph of hidradenocarcinoma showing sheets of basaloid cells with focal glandular-like structures (hematoxylin and eosin [H&E] stain, mag. 40x).

**Figure 4:**
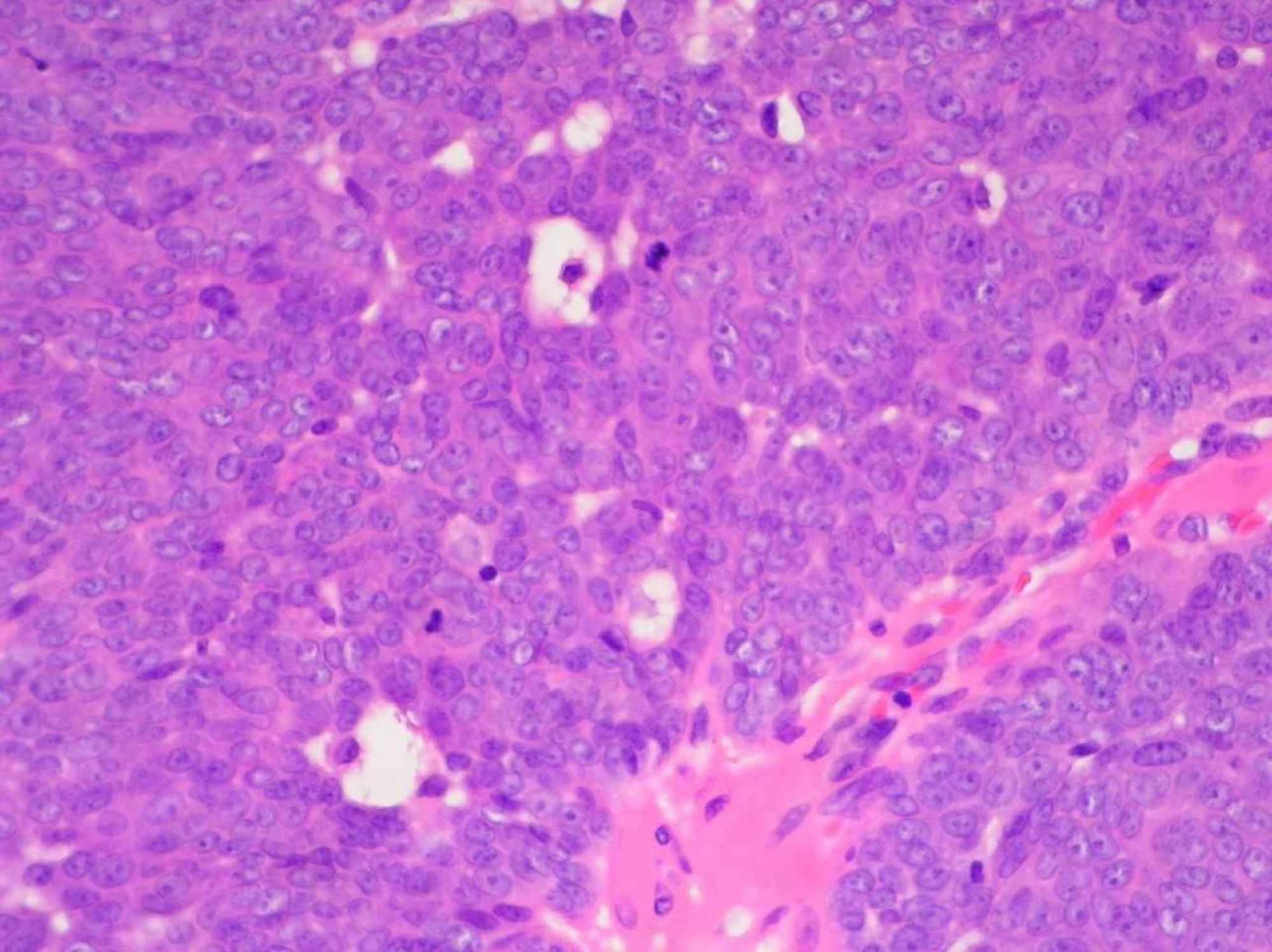
Histopathology of Tumor Specimen Mag. 60x A photomicrograph of hidradenocarcinoma showing polymorphic round to oval basaloid cells with marked atypia, distinct cell membranes, and prominent nucleoli. Some cells have clear vacuolated spaces. There is an increase in mitotic activity (hematoxylin and eosin [H&E] stain, mag. 60x).

On surgical consultation, the mass was believed to be a capillary hemangioma vs. cyst based on pre-operative clinical assessment. The mass was excised under local anesthesia and submitted to pathology. The [VZ1] specimen was notably friable during sectioning. On analysis, the specimen measured 1.2 x 0.5 cm. Histologically, the mass had a slightly irregular, lobulated, infiltrative architecture composed of a mostly solid epithelial neoplasm infiltrating the dermis with peripheral invasion. The infiltration approached the surgically inked margin. The tumor cells were arranged in sheets composed of basaloid cells with round to oval nuclei with some polymorphism and atypia, distinct cell membranes, and prominent nucleoli. Focal areas of clear cells with vacuolated cytoplasm were present. Focal areas of gland-like structures were present. There was an increase in mitotic activity. Necrosis and lymphovascular invasion were not evident. These histological findings are evident in Figures 1-4. Upon immunohistochemical staining, the tumor stained positive for pancytokeratin, cytokeratin 5/6, Ki-67, S-100, and androgen receptor. The tumor stained negative for HER-2/neu, CK7, CK20, EMA, CAM 5.2, synaptophysin, CD56, SOX-10, LCA, TTF-1, chromogranin, and CEA. EGFR staining was not performed. These histological features and stains supported the diagnosis of hidradenocarcinoma, with a recommendation of wide local excision of the primary site, sentinel lymph node biopsy, and appropriate post-operative management.

A second procedure was subsequently performed five weeks later with 3 cm margins circumferentially around the primary site and down to the pectoralis fascia (see Figure 2). Excision of a deep axillary sentinel lymph node was also performed upon detection via lymphoscintigraphy and a mapping probe. Both the wide excisional specimen and lymph node were sent to pathology. On analysis, there was no residual hidradenocarcinoma at the prior biopsy site. The lymph node was found to be negative for metastatic disease. Due to the high risk of local recurrence and malignant nature of the tumor, continued management was discussed with the hospital tumor board and oncology department. A comprehensive cancer center was also consulted to help plan the adjuvant treatment for this case. A whole-body 18F-fluorodeoxyglucose-positron emission tomography scan was performed to uncover possible metastatic disease, the results of which were negative. Adjuvant radiation therapy at carcinoma doses of 60 Gy was scheduled for local control totaling 30 treatments. Adjuvant chemotherapy is not being incorporated in the treatment regimen at this time, but anti-androgen therapy is being pursued for continued management due to androgen receptor positivity. At two-week follow up after the wide marginal excision, sutures were removed, and the adjuvant regimen was initiated.

## Discussion

Hidradenocarcinoma is an exceedingly rare malignant tumor of the eccrine sweat gland. The incidence of this tumor is approximately 0.05%, and accounts for approximately 6% of all malignant eccrine tumors. In context, malignant eccrine tumors are reported to be found in 1:13,000 skin biopsies [4]. Consequently, the literature on this malignancy is scarce, with approximately 50 cases cited. Other reports refer to the neoplasm by many names, such as clear cell hidradenocarcinoma, malignant clear cell myoepithelioma, malignant clear cell hidradenoma, and clear cell eccrine carcinoma [5]. For the purposes of this report, the term hidradenocarcinoma will be used.

This tumor arises most commonly in the sixth and seventh decades of life, with females affected slightly more often than males [4]. It should be noted that cases have also been seen in children and neonates. The most common sites include the face, scalp, and axilla, although it has also been found on the anterior trunk and extremities [5]. This tumor usually arises de novo and rarely results from a pre-existing hidradenoma [1]. The most frequent presentation is a nodular mass in the dermis with or without overlying skin changes such as erythema or ulceration. There are commonly no antecedent symptoms [1]. As such, this cancer is typically diagnosed on histology rather than clinically. The differential diagnosis is wide, including hemangioma, lipoma, lymphangioma, melanoma, basal cell carcinoma, merkel cell carcinoma, squamous cell carcinoma, and other adnexal carcinomas [3]. Criteria for malignancy include high mitotic activity, atypical nuclei, and angiolymphatic or perineural invasion. The immunohistochemistry staining for hidradenocarcinoma shows strong positivity for Ki-67, p53, keratin AE1/AE3, and cytokeratin 5/6, but negativity for CEA, S-100, GCDFP-15, EMA, and bcl-2 [4]. Although it appears variable, some reports have also found positivity for HER-2/neu and for androgen receptors [3,6]. Genetics may also play a role in the development of this cancer, including mutations in EGFR, PIK3CA, AKT-1, and TP53 [7].

Upon diagnosis, the treatment of choice is wide surgical excision with 3 cm margins, as in the current patient [3]. Sentinel lymph node biopsy is recommended in guiding treatment due to the high incidence of distant metastases [3]. The five-year median survival is quite poor at 30% [2]. There is a 50% local recurrence rate, as well as a 60% metastatic rate within the first two years of diagnosis. Metastases are most commonly found in regional lymph nodes, followed by lung and then bone [1]. There is a decreased median survival from 55 to 33 months with the presence of lymph node positivity, with a further decrease to 14.5 months in the presence of distant metastases [8].

As reported in the literature, the decision is often made to manage adjuvant therapy based on grade, margin positivity, angiolymphatic invasion, and metastasis [3]. This approach parallels current guidelines for other skin carcinomas, including melanoma and squamous cell carcinoma. Radiation of 50-70 Gy has been recommended if the tumor is greater than 5 cm, positive margins less than 1 cm, and moderate-to-poor differentiation with angiolymphatic invasion [3]. One case of hidradenocarcinoma of the parotid area received 33 fractions of external beam radiotherapy for a total dose of 66 Gy. This adjuvant treatment achieved good local control at 15 months of follow-up [9]. Adjuvant chemotherapy has also been handled on a case by case basis, with its utility not yet proven [3,4,8]. Hormonal therapy may have a role in said adjuvant management, dependent on the degree of hormone receptor positivity in any given case [3]. Emphasis should be placed on the lack of clear and widely accepted guidelines in management of this malignancy, with treatment decisions often deferring to the few case reports that are available. Regarding better treatment modalities in the future, there may be promise in targeted therapies based on molecular and genetic profiles of individual tumors, such as HER-2/Neu and trastuzumab [6]. Ongoing research will be critical to the effective diagnosis and treatment of patients with hidradenocarcinoma.

## Conclusions

Hidradenocarcinoma is a rare and highly aggressive malignancy that is often addressed with surgery, radiation, and with or without chemotherapy. In patients with rare cancers such as this, emphasis should be placed on referring them to a comprehensive cancer center for continued treatment management. For our patient, the treatment regimen consisted of surgical excision, radiation, and hormonal therapy. As the literature is scarce regarding the identification and treatment of hidradenocarcinoma, this case report serves as a contribution to said body of knowledge. Further clinical research would be highly efficacious in guiding treatment decisions for future patients with this disease.
